# Decision‐making processes in artificial intelligence applications in dentomaxillofacial radiology from the perspective of Wittgenstein

**DOI:** 10.1002/kjm2.12877

**Published:** 2024-07-19

**Authors:** Sumeyye Celik Ozsoy, Samed Satir, Usame Omer Osmanoglu

**Affiliations:** ^1^ Department of Oral and Maxillofacial Radiology, Faculty of Dentistry Karamanoglu Mehmetbey University Karaman Turkey; ^2^ Department of Biostatistics, Faculty of Medicine Karamanoglu Mehmetbey University Karaman Turkey

Artificial intelligence (AI) research studies have been the subject of philosophy, and arguments such as “Chinese room” and “Turing test” have emerged in reaction to AI.^1^ In his book *Philosophical Investigations* (original name: *Philosophische Untersuchungen*), which was published a few years after Ludwig Wittgenstein's death and has serious meaning for the history of modern philosophy, Wittgenstein makes such striking observations about the relationship between human verbal and visual perception.^2^ It is accepted that the reference to the image in Wittgenstein's book has an important role in the popularity of the “rubbit‐duck illusion,” which is frequently encountered in philosophy and illustration.^3^ It is not possible to describe exactly with this figure; however, Wittgenstein's references to the fact that we perceive different images differently when viewed the same image at different times may encourage researchers to review the methodology of AI applications in dental and maxillofacial radiology.^4^

A study was planned to show how much the quality of panoramic radiography (PAN) affects the diagnostic accuracy of radiolucent lesions in the maxillary premolar apex. To avoid memory interference and bias with the scored, observers were presented PANs with censored maxillary premolar crowns, and retrospective periapical radiographs (PAR) taken from the relevant region were used to confirm lesions. Apart from the effect of PAN quality on the diagnosis of lesions at the premolar apex, an unexpected finding was obtained. Observers scored that some teeth were absent with PAN, but no lesions were present in the same teeth with PAR. It can be predicted that observers would assume that there is no lesion, if the alveolar bone trabeculation and root apex have similar homogeneity, in the situation of they could see the crowns of the teeth without censor (Figure 1).

**FIGURE 1:**
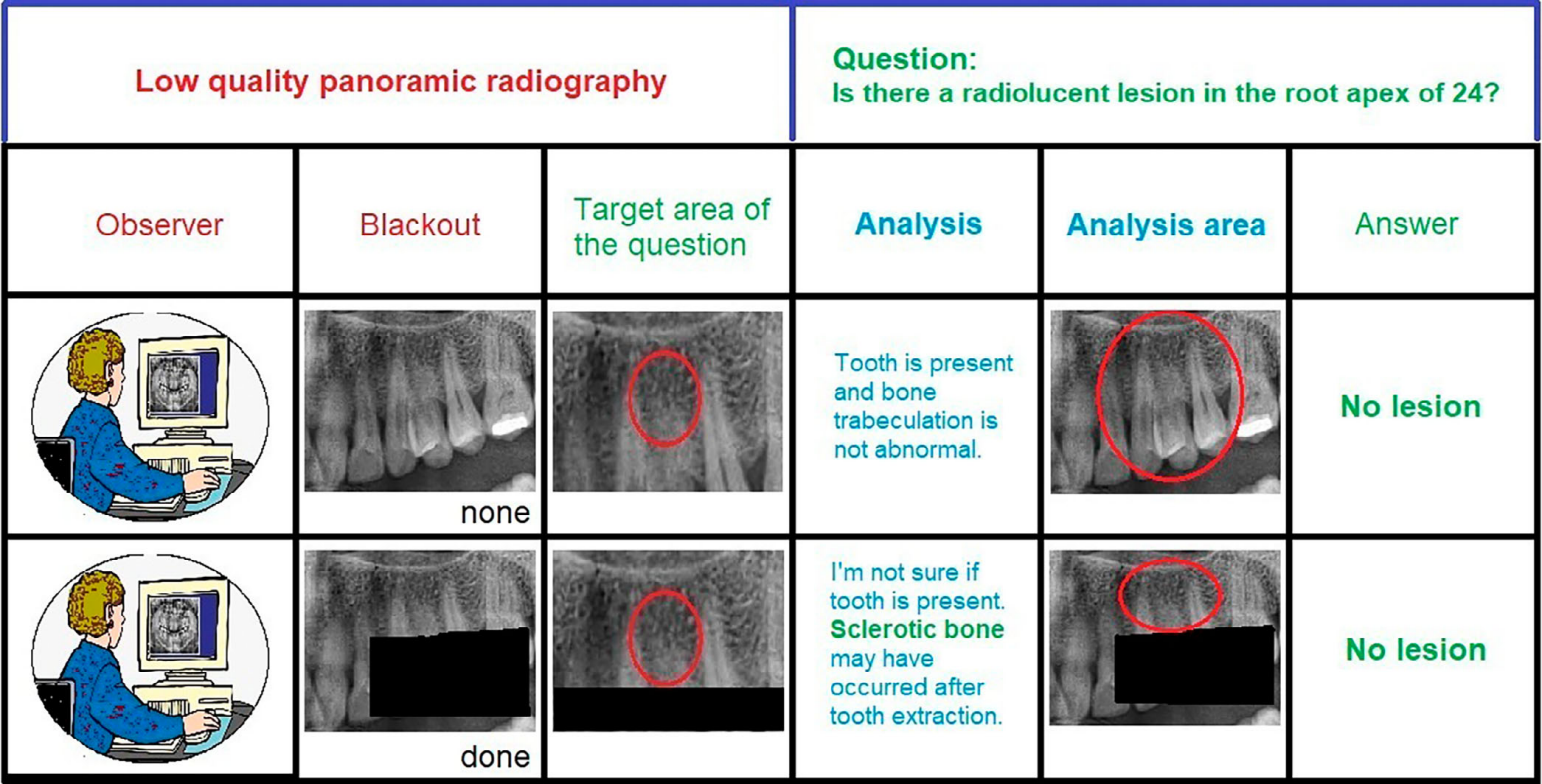
Representation of radiologists' possible evaluation and decision‐making processes.

This finding suggests that dentists/dentomaxillofacial radiologists do not only focus on the relevant area while observing the radiographs and make a final decision by associating the landmark/pathology they will detect with the surrounding anatomical structures and the patient's history. This situation may not have caused inconsistency in the results, even if the presence of the tooth was not certain in the periapical lesion scoring in study. However, it has been revealed that observer behaviors should be clarified as well as radiography quality standardization while creating the method in AI applications, which have become common in dental radiology today. In fact, the observers may not even be aware that the decision‐making mechanisms do not operate only within the limits of the guide presented in the study methodology while making the scoring. In a study in which observer behaviors and observation process are analyzed,^5^ the effect of concepts such as “focus on specific region” and “only concentrating on the area of expertise” on the accuracy of diagnosis is emphasized. It can be said that these concepts may be related to the finding of “interest region assessment error with missing visuals” in the study^5^ and that this subject should be examined with new research.

## CONFLICT OF INTEREST STATEMENT

The authors declare no conflict of interest.

## References

[1] Bishop JM . Artificial intelligence is stupid and causal reasoning will not fix it. Front Psychol. 2021;11:513474. 33584394 10.3389/fpsyg.2020.513474 PMC7874145

[2] Lüscher TF , Wenzl FA . Artificial intelligence and deep learning: Wittgenstein beats Plato. Eur Heart J. 2023;44:4403–4405. 37667826 10.1093/eurheartj/ehad576

[3] Loughlin V . Wittgenstein's challenge to enactivism. Synthese. 2021;198(Suppl 1):391–404.

[4] Hung K , Montalvao C , Tanaka R , Kawai T , Bornstein MM . The use and performance of artificial intelligence applications in dental and maxillofacial radiology: A systematic review. Dentomaxillofac Radiol. 2020;49:20190107. 31386555 10.1259/dmfr.20190107 PMC6957072

[5] Vogel D , Schulze R . Viewing patterns regarding panoramic radiographs with different pathological lesions: an eye‐tracking study. Dentomaxillofac Radiol. 2021;50:20210019. 33989018 10.1259/dmfr.20210019 PMC8611277

